# Improving Rates of Capacity Assessment in an Acute Psychiatric Ward in London

**DOI:** 10.1192/bjo.2025.10372

**Published:** 2025-06-20

**Authors:** Mia Harada-Laszlo, Sarah Elrifai, Alex Berry

**Affiliations:** 1 Whittington Hospital, London, United Kingdom; 2 Highgate Acute Mental Health Centre, London, United Kingdom

## Abstract

**Aims:** Capacity is a decision and time dependent construct and assessing capacity regularly is a core tenet of ethical practice, particularly in a psychiatric setting. However, on our ward we found that these assessments were not formally recorded for all patients. We felt it was pertinent to assess the proportion of patients for whom capacity assessments for consent to treatment and to admission were documented, and to trial interventions to improve these rates.

**Methods:** We collected retrospective data from electronic medical records of 40 patients admitted on an acute men’s psychiatric ward between 1/10/2023 and 2/2/2024. For each patient we identified whether their capacity to consent to admission or treatment was recorded on their clerking, or on any subsequent ward-round documentation. Further to this we recorded whether each patient had a capacity assessment recorded on the dedicated Rio capacity form. We then implemented changes including the circulation of a standardised proforma for ward-rounds and clerkings, which included a capacity assessment. After 6 months we re-recorded these metrics for 29 patients admitted between 15/8/2024 and 24/10/2024 and compared the results of each metric using a chi-square test.

**Results:** We found that there was an increase in the proportion of patients receiving an assessment of their capacity to consent to treatment between cycle 1 and 2. However, this did not reach statistical significance (p=0.66). Similarly, in comparing rates of assessment for capacity to consent to admission on initial clerkings, there was an increase which was not statistically significant (p=0.94). For ward-round documentation, we found an improvement in rates of capacity assessment for treatment which was not statistically significant (p=0.68), and a decrease in rates of capacity assessment for admission which was not significant (0.94). However, there was a statistically significant increase in the proportion of patients who had a formal capacity assessment documented using Rio forms (p<0.05).

**Conclusion:** We did not find any statistically significant increase in the recording of capacity assessment on doctor’s notes, either on initial clerkings or in ward-round documentation following our intervention. However, we did see a significant improvement in the use of Rio capacity forms. Notably, 24% of patients admitted during the second cycle were transfers who did not have initial clerkings on our ward, which may have confounded our results. Further interventions are therefore required in order to improve rates and specificity of capacity assessments on our ward in the future.

